# Measles Virus Hemagglutinin Protein Epitopes: The Basis of Antigenic Stability

**DOI:** 10.3390/v8080216

**Published:** 2016-08-02

**Authors:** Maino Tahara, Jean-Philippe Bürckert, Kazuhiko Kanou, Katsumi Maenaka, Claude P. Muller, Makoto Takeda

**Affiliations:** 1 Department of Virology 3, National Institute of Infectious Diseases, Musashimurayama 208-0011, Tokyo, Japan; maino@nih.go.jp; 2 Department of Infection and Immunity, Luxembourg Institute of Health, Esch-sur-Alzette L-4354, Luxembourg; Jean-Philippe.Buerckert@lih.lu (J.-P.B.); Claude.Muller@lih.lu (C.P.M.); 3 Infectious Disease Surveillance Center, National Institute of Infectious Diseases, Shinjuku-ku 162-8640, Tokyo, Japan; kanou@nih.go.jp; 4 Laboratory of Biomolecular Science, Faculty of Pharmaceutical Sciences, Hokkaido University, Sapporo 060-0812, Hokkaido, Japan; maenaka@pharm.hokudai.ac.jp

**Keywords:** measles virus, hemagglutinin protein, epitope

## Abstract

Globally eliminating measles using available vaccines is biologically feasible because the measles virus (MV) hemagglutinin (H) protein is antigenically stable. The H protein is responsible for receptor binding, and is the main target of neutralizing antibodies. The immunodominant epitope, known as the hemagglutinating and noose epitope, is located near the receptor-binding site (RBS). The RBS also contains an immunodominant epitope. Loss of receptor binding correlates with an escape from the neutralization by antibodies that target the epitope at RBS. Another neutralizing epitope is located near RBS and is shielded by an N-linked sugar in certain genotype strains. However, human sera from vaccinees and measles patients neutralized all MV strains with similar efficiencies, regardless of the N-linked sugar modification or mutations at these epitopes. Two other major epitopes exist at a distance from RBS. One has an unstructured flexible domain with a linear neutralizing epitope. When MV-H forms a tetramer (dimer of dimers), these epitopes may form the dimer-dimer interface, and one of the two epitopes may also interact with the F protein. The neutralization mechanisms of antibodies that recognize these epitopes may involve inhibiting the H-F interaction or blocking the fusion cascade after MV-H binds to its receptors.

## 1. Introduction

Measles has been a leading cause of childhood death in the past, and a significant number of measles-related deaths are still reported, mainly in developing countries. However, progress in measles control suggests that the disease can potentially be eradicated globally by vaccination [1,2]. All available data show that the measles virus (MV) is serologically monotypic. Therefore, measles vaccines that are derived from strains isolated more than 50 years ago are still highly effective against all the currently circulating MV strains. The antigenic stability of MV is one of the major factors that make global measles eradication feasible [3]. MV is classified in the genus *Morbillivirus* in the family *Paramyxoviridae*. Two types of glycoprotein spikes, the hemagglutinin (H) and fusion (F) proteins, protrude from the viral envelope. Although both the H and F proteins are the targets of neutralizing antibodies, the humoral immune response is mainly directed against the H protein [4,5,6]. The function of the H protein is to bind to a cellular receptor on the target cells. Signaling lymphocyte activation molecule (SLAM) expressed on immune cells and nectin-4 expressed on epithelial cells are receptors for MV [7,8,9,10]. In addition to SLAM and nectin-4, vaccine strains and certain laboratory strains of MV have been adapted to use CD46 as a receptor [11]. When the H protein binds to a receptor, conformational changes are triggered in the F protein, which mediate the fusion between the viral envelope and the host cell plasma membrane. The functional and physiological interactions between the H and F proteins are critical for triggering membrane fusion [12].

To date, many studies using different sets of monoclonal antibodies (MAbs) have determined the antigenic epitopes of the H protein. In general, MV is antigenically highly stable, but minor antigenic variations are caused by specific amino acid changes in the H protein [2,13,14,15,16,17]. The crystal structures of the H protein complexed with SLAM [18], nectin-4 [19], and CD46 [20] have been determined, so the antigenic epitopes or specific amino acids can be mapped onto these structures. Recent studies have also provided extensive insight into the mechanism of membrane fusion triggered by the interaction between the H and F proteins [21]. The aim of this review is to integrate the currently available data and additional data (provided in this review) to organize and maximize our understanding of the H protein epitopes and the molecular basis of the antigenic stability of MV.

In this review, the major epitopes of the H protein are classified into five types: hemagglutinating and noose epitope (HNE), receptor-binding epitope (RBE), sugar-shielded epitope (SSE), neutralizing epitope (NE), and loop epitope (LE). This review summarizes the details of the individual epitopes, including their locations and known and predicted functions, and clarifies why the MV-H protein remains antigenically stable.

## 2. Overall Structure of the H Protein

**Figure 1:**
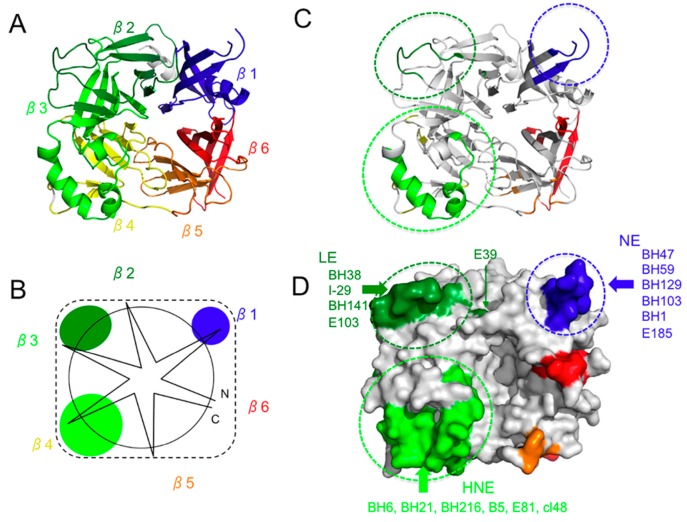

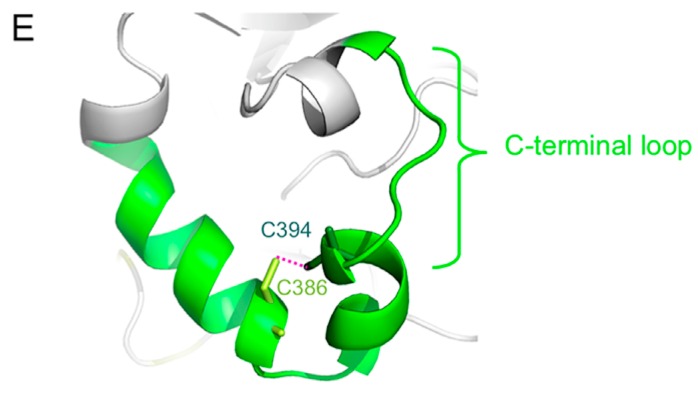
Top view of MV-H and the locations of HNE, NE, and LE. (**A**) Ribbon diagram. MV-H has a six-bladed β-propeller fold (β1–β6). β-Sheets 1, 2, 3, 4, 5, and 6 are shown in blue, red, orange, yellow, light green, and green, respectively; (**B**) Schematic diagram. Light green, blue, and green ovals indicate locations of HNE, NE, and LE, respectively. Black solid and dashed lines indicate the overall structure of the H protein; (**C**,**D**) Locations of HNE, NE, and LE. The amino acids demonstrated or suggested to constitute a portion of an epitope are shown in colors: residues on β-sheets 1, 2, 3, 4, 5, and 6 are shown in blue, red, orange, yellow, light green, and green, respectively. Light green, blue, and green dashed-line circles indicate locations of HNE, NE, and LE, respectively. (**C**) Cartoon model. (**D**) Surface presentation and MAbs that recognize HNE, NE, and LE; (**E**) Enlarged view of HNE. Stick models indicate two cysteine residues, and the dashed red line indicates the disulfide bond between the cysteines.

The head domain of the MV-H protein consists of a six-bladed β-propeller fold (β1–β6 sheets) (Figure 1A–C) [22]. When viewed from the top, the overall structure of the head domain is square, and there is an enlarged pocket in the center (Figure 1B,D) [22]. The N-linked sugar at amino position 215 (N215-sugar) is thought to shield the pocket [22]. The H protein forms a homodimer linked by an interchain disulfide bond between the cysteine residues at position 154. In the homodimer, the two H protein monomers interact with each other in a highly tilted position [22]. The dimer of the H protein is further assembled into a tetrameric structure by forming a dimer of dimers [18]. An X-ray crystal structural analysis has demonstrated two crystal forms (form I and form II) of the head domain of the MV-H protein [18]. The protein may form a different type of tetramer if it has a stalk region, and may exist in several different forms during the process of receptor binding and the subsequent triggering of membrane fusion.

## 3. Hemagglutinating and Noose Epitope (HNE)

Amino acids 379–400 form an immunodominant epitope. This epitope is known as HNE because three cysteines in the epitope form a surface-exposed loop (light green in Figure 1C,D) [23,24]. The disulfide-constrained surface-exposed loop is often described as the “noose” motif [25]. MAb-BH6, -BH21, and -BH216, which show both hemagglutination-inhibiting (HI) and -neutralizing activities, were generated after immunization with MV [23]. These antibodies recognize the conformation of HNE, and the cysteine loop formed by the disulfide bond between the cysteines at positions 386 and 394 is critical for its conformation (Figure 1E) [23]. The C-terminal loop is also important for its recognition by MAb-BH6, -BH21, and -BH216 (Figure 1E) [23]. The binding of ~40% of human serum anti-MV antibodies in convalescent measles patients and vaccinees is blocked by MAb-BH6 [26]. Although the amino acid sequence of this region is poorly conserved among the H proteins of other morbilliviruses, the two cysteines are highly conserved. Therefore, the disulfide bond may be important for the function or conformational stability of the H protein. Several previously described epitopes correspond to HNE: I recognized by MAb-B5 and MAb-E81 [27,28], E3 recognized by MAb-cl48 [29], D recognized by MAb-K71 [30], D/E recognized by MAb-NC32 [30], and F recognized by MAb-L77 [30] (Figure 1D and Table 1).

HNE is well conserved among MV strains, but certain genotype H1 strains are not neutralized by MAb-BH6 or -BH216 because they have a proline-to-leucine substitution at position 397 (P397L) [13,31]. Similarly, Japanese H1 strains with the P397L mutation are not neutralized by MAb-E81 [32]. The proline at position 397 is probably critical for MV neutralization by these MAbs. However, MAb-BH216 still binds to an HNE peptide consisting of amino acid region 379–400, even when it contains the P397L mutation [24]. These observations suggest that there are some structural differences between the HNE peptide and the native form of HNE. The sera of vaccinees efficiently neutralize these MV strains, despite the P397L mutation [13]. The roles of HNE are currently unknown, but it is probably structurally or functionally important for the H protein. A mutant that escaped neutralization by MAb-B5 and -E81 showed an attenuated capacity for replication because it had a mutation in the HNE (Q391R) [28].

MV escaped neutralization by MAb-48 when it acquired mutations at amino acid positions 395 and 398 [29]. However, MAb-48 bound to a peptide corresponding to the H protein amino acid region 126–135 (125–135 peptide) [33]. Because the affinity of MAb-48 for the peptide was lower than its affinity for MV, the 125–135 peptide probably constitutes only a portion of the natural conformational epitope [33]. The amino acid region at residues 126–135 may be located close to HNE under certain biological conditions, but their locations are currently unknown.

**Figure 2:**
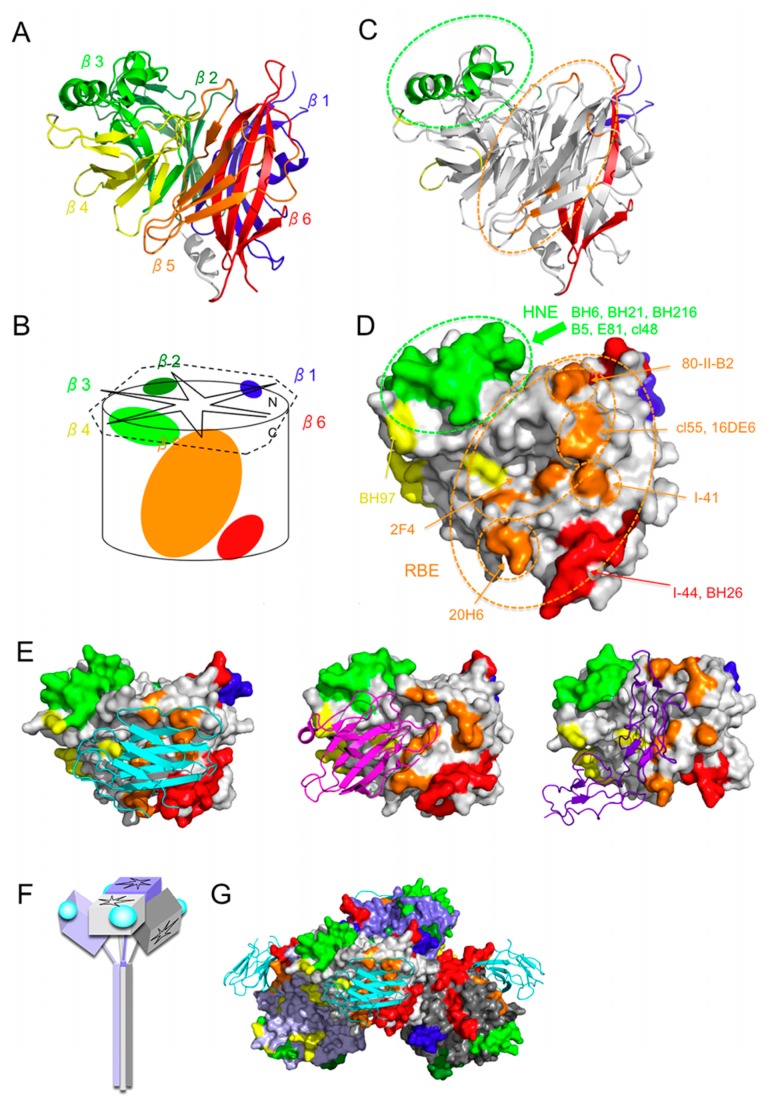
Side view showing RBE and HNE. (**A**) Ribbon diagram. The β-sheets 1, 2, 3, 4, 5, and 6 are shown in blue, red, orange, yellow, light green, and green, respectively; (**B**) Schematic diagram. Light green, blue, and green ovals indicate locations of HNE, NE, and LE, respectively. Orange and red ovals indicate the locations of RBE. Black solid and dashed lines indicate the overall structure of the H protein; (**C**,**D**) Locations of RBE and HNE. Amino acids demonstrated or suggested to constitute a portion of an epitope are shown in colors: residues on β-sheets 1, 2, 3, 4, 5, and 6 are shown in blue, red, orange, yellow, light green, and green, respectively. Orange and light green dashed-line circles indicate the locations of RBE and HNE, respectively. (**C**) Ribbon diagram. (**D**) Surface presentation and MAbs that recognize RBE and HNE; (**E**) Structures of MV-H complexed with SLAM (left), nectin-4 (middle), or CD46 (right). Surface presentation models show MV-H and cartoon models show SLAM (cyan) [18], nectin-4 (magenta) [19], and CD46 (purple) [20]; (**F**,**G**) MV-H tetramer complexed with SLAM (form I) [18]. Four H protein molecules are shown in gray, light gray, purple, and light purple. SLAM is shown in cyan. (**F**) Schematic diagram. (**G**) Surface presentation models represent MV-H and cartoon models represent SLAM [18].

MAbs that recognize HNE neutralize MV infection via SLAM, nectin-4, and CD46. MAb-B5 shows very high neutralizing titers, regardless of the receptor used by the virus (Table 2) [28]. MAb-B5 and -E81 have been shown to inhibit SLAM binding, although its inhibition by MAb-E81 is less efficient than that by MAb-B5 [28]. Both MAb-B5 and MAb-E81 also show high HI titers (Table 2). Therefore, antibodies that bind to HNE neutralize MV infection by blocking the binding of the virus to its receptor. Although HNE is not directly involved in the receptor-binding site (RBS), it is located near RBS (Figure 2). Individual MAbs may neutralize MV infection with different efficiencies when MV uses different receptors, because although the binding sites of the three receptors overlap, they still differ from one another (Figure 2E). Nectin-4 binds to a position more proximal to HNE than does SLAM or CD46, and CD46 uses a larger area for binding than SLAM or nectin-4 (Figure 2E).

## 4. Receptor-Binding Epitope (RBE)

RBS is located on the lateral side of the H protein propeller fold structure (Figure 2) [18]. A phenylalanine at position 552 interacts with SLAM [18], and a mutation at this residue causes MV to escape neutralization by MAb-I-41 (Figure 2D) [34,35]. The aspartic acids at positions 505 and 507 also interact with SLAM [18], and a mutation at position 505 or 506 allows MV to escape neutralization by MAb-80-II-B2 (Figure 2D) [5,36]. The CAM-70 vaccine strain is not neutralized by MAb-80-II-B2 because it has an amino acid substitution at position 505, and it uses SLAM less efficiently than other strains [37]. A mutant resistant to neutralization by MAb-16-DE6 has acquired a mutation at amino acid position 533, one of the residues that interact with SLAM (Figure 2D) [34,35]. A mutation at position 533 also abolishes neutralization by MAb-cl55 (Figure 2D) [38], and MV escapes neutralization by MAb-20H6 when the H protein contains mutations at positions 546 and 547 (Figure 2D) [38]. The residue at position 546 modulates the binding ability of the H protein to CD46 [11] and nectin-4 [39]. These data suggest that RBS contains a major epitope (designated “the receptor-binding epitope” [RBE]), and a mutagenesis analysis using recombinant MV provided further evidence of this. When MV was mutated at a nectin-4-interacting residue (F483A, Y543S, or Y541S), it escaped neutralization by MAb-2F4 (Figure 2D), but lost the ability to use nectin-4 as a receptor [40,41]. Similarly, MV escaped neutralization by MAb-2F4 when it mutated at a SLAM-interacting residue of the H protein (R533A or D505S) (Figure 2D), but it lost its ability to use SLAM as a receptor [40].

One study has suggested that the epitope recognized by MAb-BH26 is the most important immunodominant epitope because MAb-BH26 inhibits the binding of ~60% of human serum antibodies in convalescent measles patients and vaccinees [26]. The binding site of MAb-BH26 is predicted to be in the amino acid region 571–579 or 190–200 [5]. Both these regions constitute portions of the β6 sheet (Figure 2C), and the residues at positions 191–195 are located particularly close to RBS and interact directly with SLAM [18]. An amino acid mutation at position 190 reduces the neutralization activity of MAb-2F4 [32]. MAb-BH26 preferentially blocks MV infection via SLAM [5]. These data are consistent with the notion that the predicted BH26-recognizing region (amino acids 190–200) interacts directly with SLAM (Figure 2E) [18].

There is a linear epitope in the region defined by residues 188–199 and a potential glycosylation site at position 187 (Asn-Cys-Ser at positions 187–189) [35]. Variants that are not neutralized by MAb-I-44 have acquired an S189P or S189L mutation [35]. Therefore, the epitope recognized by MAb-I-44 may involve the N187-sugar or the linear epitope 188–199, which interacts with SLAM. A mutant protein containing N187S also lacks N187-sugar, but the mutant is neutralized efficiently by MAb-I-44 [42]. Therefore, MAb-I-44 probably recognizes amino acid sequences that include the SLAM-interacting linear epitope, rather than N187-sugar. However, biochemical analyses have indicated that MAb-I-44 only partially inhibits the H-SLAM interaction [43]. These observations suggest that N189-sugar partially shields the epitope recognized by MAb-I-44.

Various MAbs recognize RBE (Table 1). Several previously described epitopes correspond to RBE: vii recognized by MAb-2F4 [28]; III [44], E2 [29], 3 [45], and IIIB [36] recognized by MAb-16-DE6 and MAb-I-41 (Table 1). These data suggest that the capacity to escape the humoral immune response is incompatible with the functional integrity of the MV-H protein.

## 5. Epitope Shielded by N416-Sugar (Sugar-Shielded Epitope, SSE)

**Figure 3:**
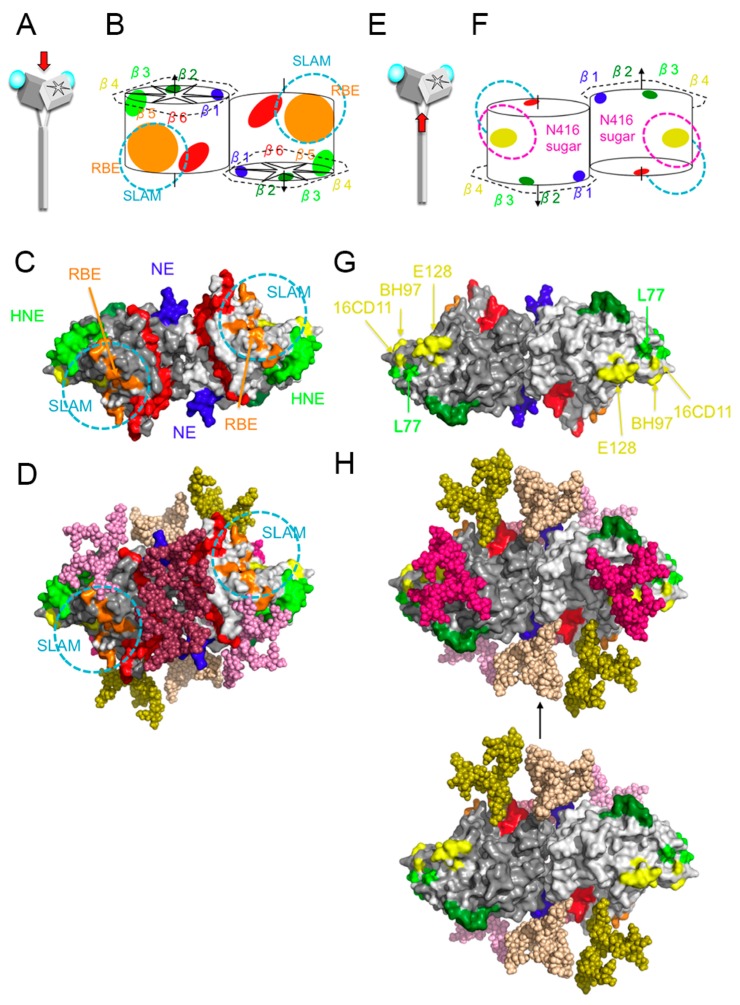
Dimer structure of the MV-H protein and models of N-linked sugar modifications. (**A**,**E**) Red arrows in (**A**) and (**E**) indicate the direction of sight for (**B**–**D**) and (**F**–**H**), respectively. (**B**,**F**) Schematic diagram. Light green, blue, and green ovals indicate locations of HNE, NE, and LE, respectively. Black solid lines and dashed lines indicate the overall structure of the H protein. Magenta and cyan dashed lines indicate locations of N416-sugar and SLAM, respectively. (**C**,**G**) Surface presentation models. Amino acids demonstrated or suggested to constitute a portion of an epitope are shown in colors: residues on β-sheets 1, 2, 3, 4, 5, and 6 are shown in blue, red, orange, yellow, light green, and green, respectively. (**D**,**H**) Surface presentation models with N-linked sugar modifications constructed with GlyProt. N-linked sugars are shown with sphere models. (**H**) Upper structure has N416-sugar and lower structure lacks N416-sugar.

The H protein is glycosylated at four amino acid positions, 168, 187, 200, and 215 [42]. Position 238 is also a potential N-linked glycosylation site, but it is not used [42]. These N-linked sugars are expected to shield a wide area of the MV-H protein, limiting the areas targeted by the humoral immune response (Figure 3) [22]. The major epitopes, including HNE and RBE, are largely unshielded by these sugars (Figure 3) [22]. Certain genotypes have acquired an additional N-linked glycosylation site at position 416 (N416-sugar) [17,46,47]. It is evident that the N416-sugar shields a specific epitope(s) (SSE) that is recognized by MAbs E128 and BH99 [28,48] (Table 1). SSE recognized by MAb-E128 contains the amino acid region 473–477 (Figure 3G) [28], so SSE may constitute a portion of the CD46-binding site [5,28,49]. The amino acids or the region recognized by MAb-BH99 are unknown, but MAb-16-CD11 competes for BH99 binding, and the epitope recognized by MAb-16CD11 contains an amino acid residue at position 491 (Figure 3G) [35]. Structural analysis data suggest that SSE does not overlap the SLAM- or nectin-4-binding site. However, MAb-E128 inhibits the SLAM-H interaction [28]. The binding of MAb-E128 also partially competes with that of MAb-2F4, which binds to the central region of RBS (Figure 2D) [40]. These findings are consistent with the observation that SSE is not RBS, but is located near RBS. A phylogenetic analysis suggested that an ancestral strain of the D3, D4, D5, D7, D8, D9, and D11 genotypes acquired the N416-sugar [50]. There are 24 genotypes [51], but only 11 have been detected in the last decade. Among these, the D4, D5, D7, D8, D9, and D11 genotype strains have N416-sugar, whereas the G3, H1 B2, B3, D6 genotype strains lack this sugar. Certain H1 genotype strains are not neutralized by MAb-E128, even though they lack the N416-sugar [32], so it is likely that they have acquired mutations in the epitope, rather than the N416-sugar shield. Both N416-sugar-possessing and -lacking strains are currently circulating worldwide. Furthermore, human sera obtained from vaccine recipients and measles patients neutralize different genotype strains with similar efficiencies, regardless of the N416-sugar modification (Table 3) [17,52]. Therefore, the advantage of N416-sugar is unclear.

## 6. Neutralizing Epitope (NE) and BH1-Binding Epitope

**Figure 4:**
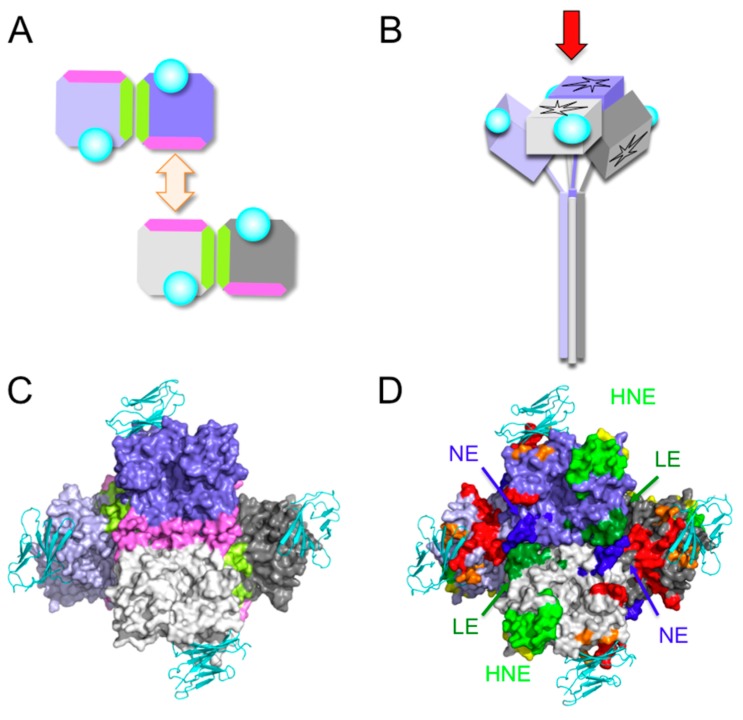
Tetrameric structure of MV-H (form I). (**A**–**D**) Four H protein molecules are shown in gray, light gray, purple, and light purple. SLAM is shown in cyan. (**A**) Schematic diagram of the formation of the dimer of dimers. Light green regions indicate the interface between the monomers in the dimer. Pink regions indicate the interface between dimers in the dimer of dimers. Pink regions of the purple and light gray MV-H molecules form the interface, whereas those of the gray and light purple MV-H molecules are exposed outside the dimer of dimers (tetramer). (**B**) Schematic diagram of the dimer of dimers (form I). Red arrow indicates the direction of sight for C and D. (**C**,**D**) Surface presentation models show MV-H, and ribbon diagrams show SLAM. (**C**) Light green regions indicate the interface between the monomers forming the dimer. Pink regions indicate the interface between the dimers forming the dimer of dimers. (**D**) Same angle as **C**. Amino acids demonstrated or suggested to constitute a portion of an epitope are shown in colors: residues on β-sheets 1, 2, 3, 4, 5, and 6 are shown in blue, red, orange, yellow, light green, and green, respectively.

Neutralizing antibodies often recognize conformational epitopes, but linear epitopes can also be targeted. MAb-BH47, -BH59, -BH103, and -BH129 recognize a linear epitope consisting of amino acids 244–250 (blue in Figure 1C,D) [53], which is designated ‘the neutralizing epitope’ (NE) [53]. The amino acid region 233–240 is recognized by MAb-BH1, and this region probably forms a surface-exposed domain together with NE, although the amino acid region at 240–247 has not been visualized in H protein structures [18,19,20,22,28], and may be an unstructured flexible region. An amino acid mutation in the BH1-binding region (E235G) severely reduces the neutralizing activities of MAb-BH47, -BH59, and -BH129, suggesting a close interaction between NE and the BH1-binding region (Table 4). MAbs recognizing NE efficiently neutralize MV infection, but show little or very low HI activity [53]. The data suggest that these MAbs do not block CD46 binding. In fact, NE is located distantly from RBS (Figure 3C). MAb-E185, which is predicted to recognize the BH1-binding region only weakly or moderately, inhibits both hemagglutination and MV infection (Table 2) [28]. MAb-BH1 binds to peptides containing the amino acid region 233–240, but binds poorly to the native form of the H protein [53]. Therefore, the BH1-binding region may not be readily accessible in the native form of the H protein. Although MAb-BH47, -BH59, and -BH129 have little or very low HI activity, they efficiently inhibit hemolysis [53]. These observations suggest that MAb-BH47, -BH59, and -BH129 inhibit the process of membrane fusion after MV-H binds to its receptor. NE is probably involved in the formation of the tetramer. In the form I tetramer, NE constitutes a portion of the dimer-dimer interface (Figure 4) [18]. Therefore, it is possible that MAbs that recognize NE inhibit the triggering of the fusion process by interfering with the arrangement of the tetramer. However, NE may not be an immunodominant epitope because MAb-BH47 only weakly inhibits the binding of human serum antibodies in convalescent measles patients and vaccinees, compared with other MAbs such as BH26, BH6, and BH38, which recognize other epitopes [26]. Certain MV genotype H1 strains are not neutralized by MAb-BH47, which recognizes NE [13].

## 7. Epitope on the Loop Protruding from the β2 Sheet (Loop Epitope; LE)

**Figure 5:**
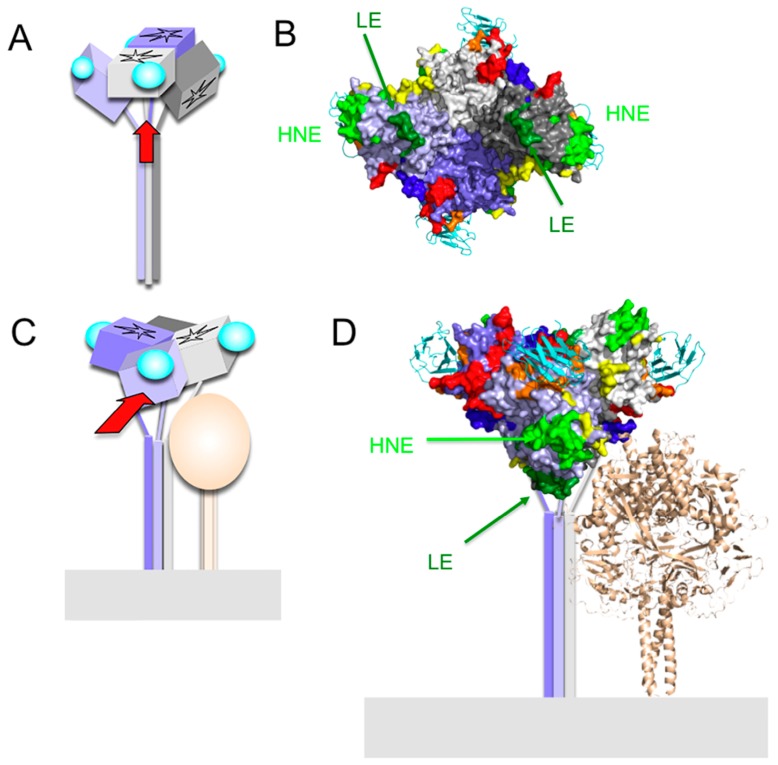
Predicted positional relationship between the MV-H tetramer (form I) and MV-F. (**A**–**D**) Four H protein molecules are shown in gray, light gray, purple, and light purple. SLAM is shown in cyan. (**C**,**D**) MV-F is shown in light brown. (**A**,**C**) Schematic diagrams of the MV-H tetramer (form I) and MV-F. Red arrows in A and C indicate the direction of sight in B and D, respectively. (**B**,**D**) Surface presentation models of MV-H. Ribbon diagrams show SLAM (cyan) and MV-F (light brown). The structural model of MV-F has been reported previously [59].

MAb-I-29 recognizes the amino acid region 309–318 and neutralizes MV [33]. A large loop protruding from the β2 sheet constitutes the epitope of this MAb, and is referred to as “the loop epitope” (LE) in this review (green in Figure 1C,D). MAb-BH38, -BH141, and -E103 recognize LE (Figure 1D), and previously described epitopes I [44], E4 [29], IA [36], 1 [45], and vi [28] correspond to this epitope (Table 1). MAb-I-29 shows little HI activity, suggesting that it does not inhibit CD46 binding, although it shows strong hemolysis-inhibiting activity [33]. This failure to inhibit receptor binding is consistent with the observation that LE is located at a position distant from RBS (Figure 2B). MAb-E103 also does not inhibit the H-SLAM interaction [28]. However, it shows some HI activity (Table 2). Therefore, MAb-E103 may inhibit the CD46 interaction. However, it also displays neutralizing activity by interfering with the H-F interaction [28]. The head domain of the H protein is situated on a stalk region, which has been shown to interact with the F protein and to play a key role in triggering fusion (Figure 5C,D) [54,55]. The crystal structure of the H protein (form I) [18] suggests that in two of the four H protein molecules (gray and light purple molecules in Figure 5), LE is located at the bottom of the tetramer. Therefore, LE may interact directly with the F protein in a higher-order structure of the H-F oligomeric protein (Figure 5D) [54,56,57]. Furthermore, LE in the other two H molecules (light gray and purple molecules in Figure 5) interacts with NE at the dimer-dimer interface of the tetramer [18] (Figure 4). Therefore, MAb-I-29 and -BH38, which recognize LE, interfere with the dimer-dimer interaction of the H tetramer [58]. These two interacting roles may generate the structural constraints against changes in LE. Supporting this, a mutant viral strain with a mutation in LE (Q311R) replicates less efficiently than the parental strain [28].

## 8. Materials and Methods

### 8.1. Cells

II-18 and B95a cells were maintained in RPMI medium (Invitrogen, Carlsbad, CA, USA) supplemented with 7.5% fetal calf serum (FCS). Vero and Vero/hNectin4 cells [60] (Vero cells constitutively expressing human SLAM and nectin-4, respectively) were maintained in DMEM (Gibco, Carlsbad, CA, USA) supplemented with 7.5% FCS.

### 8.2. Viruses

All MV strains used in the present study were recombinant viruses expressing *Renilla* luciferase [61]. The “D3” virus has the genome of the wild-type IC323 strain (genotype D3) [28,62]. The “A” virus has the genome of the wild-type IC323 strain, except for the H gene, which was derived from the Edmonston vaccine strain (genotype A) [28]. The D3/Q391R virus is a mutant “D3” virus with the Q391R mutation in the H protein [28]. The A/E235G virus is a mutant “A” virus with the E235G mutation in the H protein [28].

### 8.3. MAbs and Human Sera

Mouse MAb-E81, -B5, -E128, -E103, and -E185 (mouse ascites) have been reported previously [28]. Mouse MAb-BH47, -BH59, and -BH129 (mouse ascites) have also been reported previously [53]. Sera from patients suffering from acute measles were collected in Tokyo in 2002. Vaccinated sera were collected from one-year-old children who had received one dose of a combined measles/mumps/rubella vaccine. This study was performed with the approval of the Ethics Committee of the National Institute of Infectious Diseases, Japan.

### 8.4. Neutralizing Assay

The details of the method have been reported previously [28]. Briefly, 2000 PFU of recombinant MV was incubated with serially diluted MAbs or human sera, then inoculated into the culture medium for II-18, B95a, Vero, or Vero/hNectin4 cells. At two days post-infection, the luciferase activity in the cells was measured. The neutralizing titer was indicated by the maximum dilution causing a >50% reduction in luciferase activity.

### 8.5. HI Assay

MV antigen (4 hemagglutinating units in a volume of 25 µL) was added to 25 µL of serially twofold-diluted MAbs in U-bottom 96-well microplates. Phosphate-buffered saline (PBS; pH 7.2) containing 0.1% bovine serum albumin and 0.01% gelatin was used for dilution. Each well received 50 µL of a 0.5% suspension of African green monkey erythrocytes. Plates were shaken and incubated for 1.5 h at 37 °C. The highest dilution of each MAb that completely inhibited hemagglutination was considered the HI titer of that MAb.

### 8.6. Structures of H and F Proteins

The crystal structures of the H protein unbound to its receptors [22] and when complexed with SLAM [18], nectin-4 [19], or CD46 have been reported previously [20]. The model structure of the MV-F protein was constructed based on the previously reported crystal structure of the F protein of parainfluenza virus type 5 (PDB: 2B9B) [59]. Sugar modifications on the H protein were modeled with GlyProt (http://www.glycosciences.de/modeling/glyprot/php/main.php). Figures were generated with PyMOL (http://www.pymol.org).

## 9. Conclusions

In this review, we have reorganized large amounts of data from various papers concerning the MV-H protein epitopes, together with additional data. The MV-H protein epitopes can be roughly clustered into five distinct epitopes. The first is HNE; the second comprises NE and the BH1-binding epitope; the third is LE and is located on the loop protruding from the β2 sheet; and the fourth is RBE. The exact location of the fifth epitope, SSE, is still unclear, but it is located near RBS and is shielded by the N416-sugar in the specific genotype strains currently circulating.

Among these epitopes, the function of RBE is very clear. The epitope is part of a region that must bind to two proteinaceous receptors, SLAM and nectin-4. Therefore, it is not shielded by sugars and must be subjected to strong structural constraints. Significantly, amino acid changes that allow MV to escape neutralization cause a loss of its receptor-binding activity [40]. The antibodies in polyclonal sera probably target various regions of this epitope, and it is the most important immunodominant epitope recognized by human sera [26]. Therefore, the RBE may contribute greatly to the antigenic stability of MV. The roles of NE and LE are still unclear, but it is interesting that they interact to form the dimer-dimer interface in a certain form of the MV-H tetramer. LE may also interact with the F protein. These epitopes may be involved in a step(s) in the membrane fusion cascade, mediated co-operatively by the H and F proteins. The structural and functional roles in the protein-protein interactions may constitute structural constraints against change to these epitopes. HNE is the best-known epitope of the MV-H protein, although its function is still unclear. Human sera recognize this epitope as an immunodominant epitope [26], and it must have an important role because its overall structure is conserved among the morbilliviruses. The epitope may be critical for the structural stability of the MV-H protein or it may function in the interactions between the multiple units of the H protein dimers and F protein trimers.

Currently used MV vaccines were generated from MV strains isolated more than 50 years ago. Nonetheless, they are still highly effective against the MV strains circulating at present. Sera obtained from convalescent measles patients and vaccinees neutralize different MV strains, including vaccine strains and currently circulating strains, with similar efficiencies. This fact and our knowledge of the H protein epitopes strongly suggest that MV cannot undergo large antigenic changes, so the global elimination of MV should be feasible with the tools at hand. This comprehensive review of the epitopes of the MV-H protein will contribute to the global efforts to this end.

## Figures and Tables

**Table 1:** Relationships between epitopes and MAbs.

	Amino Acids ^1^	MAb		Epitope Name							
β1	235	E185				iv					
	233–240	BH1									
	235, 244–250	BH47, BH59, BH103, BH129		NE							
β2	302	E39				v					
	310	BH38	LE					E4			
	310	BH141	LE					E4			
	309–318	I-29	LE				I	E4		IA	1
	n.d. ^2^	I-12	LE				I			IA	1
	311	E103	LE			vi					
β3	377–378	L77							F		
	391	B5			I						
	391	E81			I						
	379–400	BH6, BH21, BH216		HNE							
	395, 398	cl48						E3			
	n.d.	K71							D		
	n.d.	NC32							D/E		
β4	473–477	E128	SSE		II						
	491	16-CD11	SSE				II			II	2
	n.d.	BH99	SSE								
	488	BH97									
	483	2F4	RBE			vii					
β5	505, 541, 543, 533	2F4	RBE			vii					
	505, 506	80-II-B2	RBE								
	533	cl55	RBE								
	532, 533	16-DE6	RBE				III	E2			3
	547, 546	20H6	RBE								
	552	I-41	RBE				III	E2		IIIB	3
β6	187	I-44	RBE				IV			IV	4
	190	2F4	RBE			vii					
	190–200, 571–579	BH26									
References ^3^			This	23	27	28	31	29	30	32	33
			review	34		35					

^1^ Amino acids demonstrated or suggested to constitute a portion of an epitope; ^2^ n.d., not determined; ^3^ Corresponds to the epitope name.

**Table 2:** Neutralizing titers and hemagglutination-inhibiting (HI) titers of MAbs.

	Neutralizing Titer											HI
Cell		B95a				II-18				Vero		RBC ^3^
Receptor		SLAM				Nectin-4				CD46		
Virus ^1^		A	D3	D3/Q391R		A	D3	D3/Q391R		A		
MAb ^2^	E81	2560	5120	<40		81,920	81,920	<1280		163,840		40,960
	B5	81,920	20,480	<1280		81,920	10,240	<1280		163,840		20,480
	E128	5120	40	n.d. ^4^		163,840	<2560	n.d.		655,360		40,960
	E103	2560	10,240	20,480		10,240	20,480	20,480		20,480		2560
	E185	640	40	n.d.		n.d.	n.d.	n.d.		2560		2560

^1^ Viruses cited in this table have been reported previously [28]; ^2^ Undiluted ascites containing MAb; ^3^ Red blood cells of African green monkey; ^4^ n.d., not determined.

**Table 3:** Neutralizing titers of human sera from five vaccine recipients and four measles patients ^1^.

Virus ^2^	MAb ^3^		Vaccinees						Measles Patients			
	E128		#v9	#v12	#v14	#v27	#v29		#p1	#p2	#p3	#p4
A	163,840		80	640	160	80	80		40	40	320	5120
D3	<2560		160	640	160	80	160		320	160	640	20,480

^1^ Data in II-18 cells; ^2^ Viruses used in this table have been reported previously [28]; ^3^ Undiluted ascites containing MAb.

**Table 4:** Neutralizing titers of MAbs that recognize NE ^1^.

Virus ^2^		A	A/E235G	D3
MAb ^3^	BH47	81,920	10,240	<640
	BH59	20,480	1280	<160
	BH129	20,480	640	<160

^1^ Data in Vero/hNectin4 cells; ^2^ Viruses used in this table have been reported previously [28]; ^3^ Undiluted ascites containing MAb.
