# Erratum: Tahara, M., et al. Measles Virus Hemagglutinin Protein Epitopes: The Basis of Antigenic Stability. *Viruses* 2016, *8*, 216

**DOI:** 10.3390/v8110313

**Published:** 2016-11-21

**Authors:** Maino Tahara, Jean-Philippe Bürckert, Kazuhiko Kanou, Katsumi Maenaka, Claude P. Muller, Makoto Takeda

**Affiliations:** 1 Department of Virology 3, National Institute of Infectious Diseases, Musashimurayama 208-0011, Tokyo, Japan; maino@nih.go.jp; 2 Department of Infection and Immunity, Luxembourg Institute of Health, Esch-sur-Alzette L-4354, Luxembourg; Jean-Philippe.Buerckert@lih.lu (J.-P.B.); Claude.Muller@lih.lu (C.P.M.); 3 Infectious Disease Surveillance Center, National Institute of Infectious Diseases, Shinjuku-ku 162-8640, Tokyo, Japan; kanou@nih.go.jp; 4 Laboratory of Biomolecular Science, Faculty of Pharmaceutical Sciences, Hokkaido University, Sapporo 060-0812, Hokkaido, Japan; maenaka@pharm.hokud

The authors wish to make the following change to their paper [1].

Table 1 should be replaced with:

The authors apologize for any inconvenience this may cause.

The change does not affect the scientific results. The manuscript will be updated and the original will remain online on the article webpage.

## Figures and Tables

**Table 1:** Relationships between epitopes and MAbs.

	Amino Acids ^1^	MAb		Epitope Name							
β1	235	E185				iv					
	233–240	BH1									
	235, 244–250	BH47, BH59, BH103, BH129		NE							
β2	302	E39				v					
	310	BH38	LE					E4			
	310	BH141	LE					E4			
	309–318	I-29	LE				I	E4		IA	1
	n.d. ^2^	I-12	LE				I			IA	1
	311	E103	LE			vi					
β3	377–378	L77							F		
	391	B5			I						
	391	E81			I						
	379–400	BH6, BH21, BH216		HNE							
	395, 398	cl48						E3			
	n.d.	K71							D		
	n.d.	NC32							D/E		
β4	473–477	E128	SSE		II						
	491	16-CD11	SSE				II			II	2
	n.d.	BH99	SSE								
	488	BH97									
	483	2F4	RBE			vii					
β5	505, 541, 543, 533	2F4	RBE			vii					
	505, 506	80-II-B2	RBE								
	533	cl55	RBE								
	532, 533	16-DE6	RBE				III	E2			3
	547, 546	20H6	RBE								
	552	I-41	RBE				III	E2		IIIB	3
β6	187	I-44	RBE				IV			IV	4
	190	2F4	RBE			vii					
	190–200, 571–579	BH26									
References ^3^			This	[23]	[27]	[28]	[44]	[29]	[30]	[36]	[45]
			review	[53]		[40]					

^1^ Amino acids demonstrated or suggested to constitute a portion of an epitope; ^2^ n.d., not determined; ^3^ Corresponds to the epitope name.
